# Association of beta-2-microglobulin, cystatin C and lipocalin-2 with stroke risk in the general Chinese population

**DOI:** 10.1080/07853890.2023.2203516

**Published:** 2023-05-08

**Authors:** Juanying Zhen, Shuyun Liu, Ryan Yan Lam Kam, Guoru Zhao, Hao Peng, Jianguo Liang, Aimin Xu, Chao Li, Lijie Ren, Jun Wu, Bernard Man Yung Cheung

**Affiliations:** a Department of Neurology, Peking University Shenzhen Hospital, Shenzhen, China; b Department of Medicine, School of Clinical Medicine, The University of Hong Kong, Queen Mary Hospital, Pokfulam, Hong Kong, China; c Department of Neurology, Shenzhen Longhua District Central Hospital, Shenzhen, China; d CAS Key Laboratory of Human-Machine Intelligence-Synergy Systems, Research Center for Neural Engineering, Shenzhen Institute of Advanced Technology, Chinese Academy of Sciences, Shenzhen, China; e Department of Epidemiology, School of Public Health, Medical College of Soochow University, Suzhou, China; f Precision Health Research Center Company Limited, Kowloon, Hong Kong, China; g State Key Laboratory of Pharmaceutical Biotechnology, The University of Hong Kong, Pokfulam, Hong Kong, China; h Department of Neurology, The First Affiliated Hospital of Shenzhen University, Shenzhen Second People’s Hospital, Shenzhen, China; i Institute of Cardiovascular Science and Medicine, The University of Hong Kong, Hong Kong, China

**Keywords:** Beta-2-microglobulin, cystatin C, lipocalin-2, stroke risk

## Abstract

**Introduction:**

Beta-2-microglobulin (B2M), cystatin C and lipocalin-2 (LCN-2) are established renal biomarkers, yet their roles in stroke have not been fully evaluated. We aimed to investigate the relationship of B2M, cystatin C, and LCN-2 with stroke risk in a general Chinese population.

**Methods:**

We used ordinal regression to study the relationship between serum B2M, cystatin C, and LCN-2 with stroke risk in 1060 participants (mean age 45.4 ± 10.8 years, 46% male) from the Shenzhen-Hong Kong United Network on Cardiovascular Disease (SHUN-CVD) study. Stroke risk was classified into low-risk, middle-risk and high-risk groups according to the China National Stroke Screening Survey criteria. Serum biomarker levels were measured using immunoturbidimetric assays. Participants with valid data on serum biomarker levels and stroke risk were included in the analysis.

**Results:**

The number of participants in the low-risk, middle-risk and high-risk stroke risk groups were 663, 143 and 254 respectively. Elevated serum B2M, cystatin C, and LCN-2 levels were associated with being male, overweight/obesity, hypertension, alcohol consumption and smoking. Serum B2M, cystatin C and LCN-2 levels were significantly associated with stroke risk in the overall population (B2M: *β* = 0.595, *p* < .001; cystatin C: *β* = 3.718, *p* < .001; LCN-2: *β* = 0.564, *p* < .001) after adjustment for age.

**Conclusion:**

Elevated serum B2M, cystatin C and LCN-2 levels are associated with stroke risk. They may be novel biomarkers for clinicians to assess stroke risk.

**Key messages**
1. Serum beta-2-microglobulin, cystatin C and lipocalin-2 levels are significantly associated with stroke risk.
2. Beta-2-microglobulin, cystatin C and lipocalin-2 may serve as useful biomarkers for stroke risk stratification in the general population.

## Introduction

Stroke is the second leading cause of death globally. According to the World Health Organization, 15 million people worldwide experience a stroke annually, of whom five million die and another five million suffer from permanent disability [1]. Thus, the discovery and validation of novel biomarkers for stroke are urgently needed. Biomarkers provide a useful tool for risk estimation, allowing the identification of those patients at high risk of stroke.

Owing to the high morbidity of cardiovascular diseases (CVD) in patients with chronic kidney disease (CKD), renal biomarkers such as beta-2-microglobulin (B2M), cystatin C and lipocalin-2 (LCN-2) have attracted increasing attention. B2M is a 100-aminoacid protein with a molecular weight of 11.8 kDa and is an important component of the major histocompatibility complex class I (MHC-I). Elevated B2M levels are found in patients with CKD, especially in dialysis patients [2]. Cystatin C is a cysteine proteinase inhibitor that can pass through the glomerular filtration membrane freely. It has a constant production rate and is recognised as an important marker for evaluating glomerular filtration rate [3]. LCN-2, which is also called neutrophil gelatinase-associated lipocalin, is a 25KDa-secreted protein in the lipocalin protein family. It is a promising biomarker that overcomes some of the shortcomings of the use of serum creatinine for diagnosing acute renal injury (AKI) [4].

Currently, the associations between these renal biomarkers and stroke have not been systematically assessed. First of all, while a number of studies have observed a significant association between B2M and both inflammation [5] and CVD [6], the role of B2M in stroke is still unclear. Second, although a previous meta-analysis reported a significant association between cystatin C and CVD incidence [7], recent studies have shown that cystatin C is not causally related to CVD in the general population [8,9]. However, animal models have indicated that cystatin C may play a neuroprotective role in stroke [10]. Therefore, there is a need to investigate the relationship between cystatin C and stroke in the general population. Our previous study demonstrated that LCN-2 significantly improved the prediction model for new-onset major adverse cardiovascular events in patients with coronary artery disease [11]. Previous studies with small sample sizes showed that LCN-2 was implicated in stroke in patients with renal dysfunction [12,13]. Therefore, a larger sample size can better evaluate the relationship between LCN-2 and stroke in the general population.

To address the above important research gaps, this study aimed to investigate the association of B2M, cystatin C and LCN-2 with stroke risk in the general Chinese population.

## Methods

### Study population

We analysed data from the Shenzhen-Hong Kong United Network on Cardiovascular Disease (SHUN-CVD) study, which is a population-based study that began in China in December 2020. To date, 3400 participants from different communities in Shenzhen have been enrolled. The assessment included face-to-face interviews, physical examinations and laboratory tests. Standardized questionnaires were used to obtain participants’ demographic data, lifestyle factors, medical history, drug history and family history. Blood pressure, height, and weight were measured during the physical examination. Blood samples were taken from the initial group of participants for the measurement of biomarkers including B2M, cystatin C, and LCN-2 after fasting for eight hours. The study was reviewed and approved by the institutional review board of Peking University Shenzhen Hospital and the University of Hong Kong. All participants gave written informed consent.

Participants with valid data on serum biomarker levels, stroke risk, smoking, alcohol consumption, physical activity, BMI, hypertension, hypercholesterolemia and diabetes were included in the analysis.

### Study outcome

Stroke risk was classified as low-risk, middle-risk and high-risk groups according to the China National Stroke Screening Survey criteria [14,15]. The risk factors of stroke included hypertension, hypercholesterolaemia, diabetes, atrial fibrillation, valvular heart disease, smoking, obesity, physical activity, family history of stroke, history of transient ischemic attack and history of stroke. The high-risk group was defined as participants with three or more stroke risk factors. The middle-risk group was defined as those with fewer than three risk factors, but at least one of the following chronic diseases: hypertension, diabetes, atrial fibrillation, or valvular heart disease. The low-risk group was defined as those with fewer than three risk factors and none of the chronic diseases listed above.

### Clinical assessment

Smoking was defined as smoking any tobacco product daily or occasionally or having a history of smoking. Alcohol consumption was defined as consuming any type of alcoholic beverage (e.g. beer, wine, liquor) at least once a week. Being physically active was defined as participating in 30 or more minutes of moderate or vigorous activity three days per week. BMI was calculated by dividing participants’ body weight (in kilograms) by their height (in meters squared). BMI ≥ 25 kg/m^2^ was considered overweight/obese. Blood pressure was measured after a 15-minute rest in the right arm at the heart level with participants seated with feet flat on the floor and back supported. Hypertension was defined as the self-reported previous diagnosis, average blood pressure at or above 140/90 mmHg, or taking antihypertensive medication. Hypercholesterolaemia was defined as the self-reported previous diagnosis, total serum cholesterol level ≥ 5.2 mmol/L or taking medication for hypercholesterolaemia. Diabetes was defined as the previous diagnosis of diabetes, fasting plasma glucose level ≥ 7.0 mmol/L or taking medication for diabetes.

### Blood sample collection and biomarker measurement

All blood samples were obtained after fasting for eight hours. The blood samples were immediately processed and stored at −80 °C or below until assayed. B2M, cystatin C and LCN-2 were measured in serum samples using an immunoturbidimetric assay. Samples were added and mixed with the reaction buffer in a cuvette to incubate. Then, the test reagent (a suspension of microparticles coated with biomarker antibodies) was added to the cuvette and mixed. The aggregation of immune particles was quantified by absorbance (main wavelength: 570 nm). The optimal levels of sensitivity and low interference are achieved at a wavelength of 570 nm. The biomarker levels were calculated by interpolation from the reference curve. Detailed procedures can be found on the website listed here: https://www.immunodiagnostics.com.hk/.

### Statistical analysis

We compared the baseline characteristics of the participants using Chi-square or one-way analyses of variance (ANOVA) as appropriate. Pearson correlation coefficients were used to examine the correlations across the biomarkers. Results are shown as mean ± standard deviation or number (percent). The LCN-2 levels showed a log-normal distribution, so we applied a natural logarithmic transformation to the data before analysis. Ordinal logistic regression was used to investigate the relationship between serum biomarker level and stroke risk. *p* ≤ .05 was considered statistically significant. Statistical analysis was performed by SPSS software Version 27 (IBM Corporation, Armonk, New York).

## Results

Of the 3400 eligible participants aged above 18 years, 1060 participants (485 men and 575 women) with a mean age of 45.4 ± 10.8 years were included in the analysis (Supplementary Figure 1).

**Figure 1.**
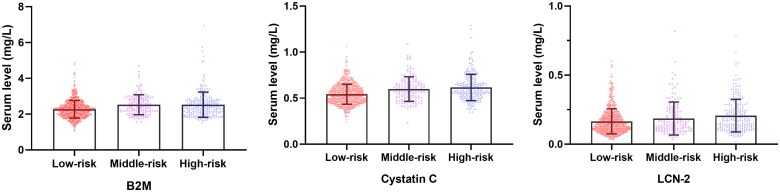
Serum biomarkers levels in different stroke risk groups. B2M, beta-2-microglobulin; LCN-2, lipocalin 2.

The clinical characteristics of participants in different stroke risk groups are shown in Table 1. The number of participants in the low-risk, middle-risk and high-risk groups were 663, 143 and 254, respectively. Compared with participants in the low-risk group, participants in the high-risk group were more likely to be older, men, smokers, physically inactive and have hypercholesterolaemia. Figure 1 shows the serum levels of biomarkers in different stroke risk groups. Elevated biomarker levels were found in the high-risk group (2.53 ± 0.71 mg/L for B2M, 0.62 ± 0.14 mg/L for cystatin C, 0.21 ± 0.13 mg/L for LCN-2; *p* < .001).

**Table 1.** Clinical characteristics of participants in the three stroke risk groups.

	Stroke risk groups			
	Low-risk	Middle-risk	High-risk	*p*
*N*	663	143	254	
Age, years	43.6 ± 10.0	51.4 ± 9.7	46.3 ± 9.8	<.001
Men	238 (35.9)	64 (44.8)	183 (72.0)	<.001
BMI, kg/m^2^	22.6 ± 3.0	23.7 ± 3.0	26.4 ± 3.9	<.001
SBP, mmHg^a^	112.3 ± 11.9	134.4 ± 14.2	129.2 ± 18.1	<.001
DBP, mmHg^a^	74.5 ± 7.9	87.8 ± 8.7	85.5 ± 11.2	<.001
Smoking	57 (8.6)	7 (4.9)	86 (33.9)	<.001
Physical activity	323 (48.7)	100 (69.9)	70 (27.6)	<.001
Alcohol consumption	23 (3.5)	7 (4.9)	44 (17.3)	<.001
Hypercholesterolaemia	183 (27.6)	36 (25.2)	168 (66.1)	<.001
Fasting plasma glucose, mmol/L	4.42 ± 0.48	5.27 ± 1.95	4.95 ± 1.55	<.001
B2M, mg/L	2.28 ± 0.50	2.52 ± 0.56	2.53 ± 0.71	<.001
Cystatin C, mg/L	0.54 ± 0.11	0.60 ± 0.13	0.62 ± 0.14	<.001
LCN-2, mg/L	0.17 ± 0.11	0.19 ± 0.14	0.21 ± 0.13	<.001

Data are presented as mean ± standard deviation or number (percent).

BMI: body mass index; SBP: systolic blood pressure; DBP: diastolic blood pressure; B2M: beta-2-microglobulin; LCN-2: lipocalin 2.

^a^70 subjects taking anti-hypertensive medication were excluded from the analysis.

Levels of B2M, cystatin C and LCN-2 among different variables are shown in Table 2. B2M and cystatin C were associated with age (*r* = 0.398 for B2M, *r* = 0.386 for cystatin C, both *p* < .001) but the association was not significant between LCN-2 and age (*p* = .183). B2M, cystatin C and LCN-2 were all significantly associated with sex, overweight/obesity, hypertension, alcohol consumption and smoking. Cystatin C was associated with physical activity. There was a significant association of B2M and cystatin C with hypercholesterolaemia (B2M: *r* = 0.065, *p* = .034; cystatin C: *r* = 0.115, *p* < .001). B2M was associated with diabetes (*r* = 0.107, *p* < .001). B2M, cystatin C and log-transformed LCN-2 were correlated with each other after adjusting for sex and age (*p* < .001) (Supplementary Table 1).

**Table 2.** Levels of B2M, cystatin C and LCN-2 among different strata of stroke risk factors.

	B2M, mg/L		Cystatin C, mg/L		LCN-2, mg/L	
	Mean ± SD	*p*	Mean ± SD	*p*	Mean ± SD	*p*
Age		<.001		<.001		.183
18–39	2.12 ± 0.44		0.51 ± 0.11		0.17 ± 0.09	
40–59	2.44 ± 0.55		0.58 ± 0.12		0.19 ± 0.13	
>60	2.89 ± 0.78		0.67 ± 0.12		0.18 ± 0.11	
Sex		<.001		<.001		<.001
Men	2.51 ± 0.60		0.63 ± 0.12		0.21 ± 0.13	
Women	2.25 ± 0.53		0.52 ± 0.10		0.16 ± 0.10	
Overweight/obesity		.002		<.001		.019
No	2.33 ± 0.58		0.56 ± 0.12		0.18 ± 0.11	
Yes	2.45 ± 0.54		0.59 ± 0.12		0.19 ± 0.13	
Hypertension		<.001		<.001		<.001
No	2.30 ± 0.52		0.55 ± 0.11		0.17 ± 0.11	
Yes	2.62 ± 0.59		0.53 ± 0.15		0.21 ± 0.15	
Smoking		<.001		<.001		<.001
No	2.34 ± 0.57		0.56 ± 0.12		0.18 ± 0.12	
Yes	2.57 ± 0.56		0.65 ± 0.10		0.22 ± 0.12	
Physical activity		.075		.020		.657
Inactive	2.34 ± 0.59		0.56 ± 0.13		0.18 ± 0.12	
Active	2.40 ± 0.56		0.58 ± 0.12		0.18 ± 0.12	
Alcohol consumption		.030		<.001		.007
No	2.36 ± 0.57		0.56 ± 0.13		0.18 ± 0.12	
Yes	2.51 ± 0.57		0.63 ± 0.10		0.22 ± 0.14	
Hypercholesterolaemia		.034		<.001		.449
No	2.34 ± 0.55		0.56 ± 0.12		0.18 ± 0.12	
Yes	2.42 ± 0.61		0.59 ± 0.13		0.19 ± 0.12	
Diabetes		<.001		.107		.094
No	2.36 ± 0.55		0.57 ± 0.12		0.18 ± 0.11	
Yes	2.67 ± 0.98		0.60 ± 0.15		0.21 ± 0.19	

B2M: beta-2-microglobulin; LCN-2: lipocalin 2.

Table 3 shows that the association between serum B2M level and stroke risk was significant in the overall population (*B* = 0.726, 95% CI = 0.506–0.947, *p* < .001), men (*B* = 0.289, 95% CI = 0.001–0.576, *p* = .049) and women (*B* = 0.928, 95% CI = 0.569–1.287, *p* < .001). After adjusting for age, the association was significant in the overall population (*B* = 0.585, 95% CI = 0.337–0.809, *p* < .001) and women (*B* = 0.445, 95% CI = 0.046–0.845, *p* = .046) but not in men (*B* = 0.254, 95% CI = −0.066–0.542, *p* = .095).

**Table 3.** The association of renal biomarkers with stroke risk in participants.

Stratum	Unadjusted *β*	95% CI	*p*	Adjusted *β*^a^	95%CI	*p*
B2M						
Overall	0.726	0.506–0.947	<.001	0.595	0.337–0.809	<.001
Men	0.289	0.001–0.576	.049	0.254	−0.066–0.542	.095
Women	0.928	0.569–1.287	<.001	0.445	0.046–0.845	.046
Cystatin C						
Overall	4.205	3.180–5.230	<.001	3.718	2.561–4.745	<.001
Men	1.872	0.465–3.278	.009	1.739	0.208–3.159	.019
Women	4.029	2.252–5.806	<.001	0.744	−1.480–2.968	.512
log-transformed LCN-2						
Overall	0.561	0.335–0.786	<.001	0.564	0.323–0.779	<.001
Men	0.399	0.087–0.711	.012	0.394	0.068–0.694	.013
Women	0.292	−0.061–0.646	.105	0.315	−0.050–0.681	.091

^a^Adjusted for age.

To find out if cystatin C can be a potential biomarker of stroke, we analysed the association between serum cystatin C level and stroke risk (Table 3). Higher cystatin C levels were significantly associated with stroke risk in the overall population (*B* = 4.205, 95% CI = 3.180–5.230, *p* < .001), in men (*B* = 1.872, 95% CI = 0.465–3.278, *p* = .009) and in women (*B* = 4.029, 95% CI = 2.252–5.806, *p* < .001). After adjusting for age, the association was significant in the overall population (*B* = 3.718, 95% CI = 2.561–4.745, *p* < .001), men (*B* = 1.739, 95% CI = 0.208–3.159, *p* = .019) but not in women.

As shown in Table 3, log-transformed LCN-2 was significantly associated with stroke risk in the overall population (*B* = 0.561, 95% CI = 0.335–0.786, *p* < .001) and men (*B* = 0.399, 95% CI = 0.087–0.711, *p* = .012). After adjusting for age, the association remained significant in the overall population (*B* = 0.564, 95% CI = 0.323–0.779, *p* < .001) and men (*B* = 0.394, 95% CI = 0.068–0.694, *p* = .013). There was no association between log-transformed LCN-2 and stroke risk in women in any of the models.

## Discussion

In this population-based study of adults aged above 18 years, we confirmed that there is a significant association of serum B2M, cystatin C and LCN-2 levels with stroke risk in a general Chinese population, even after adjusting for age. These findings support the concept that B2M, cystatin C and LCN-2 are novel biomarkers for stroke risk.

Our study evaluated the effect of three renal biomarkers on stroke risk. Although most participants had not suffered a stroke at the time of the study, 24% of participants had a high stroke risk. The use of B2M, cystatin C and LCN-2 as possible biomarkers allows the early identification of people with high stroke risk. This is the key to promoting lifestyle changes and addressing risk factors such as hypertension to prevent stroke. The participants in this study were recruited from different communities in Shenzhen, which is a city of migrants. Therefore, the study population may be representative of the general population in China, at least in terms of ethnic origin and genetics.

B2M, cystatin C and LCN-2 are biomarkers of slightly different aspects of renal function. B2M is a biomarker of tubular function. As B2M normally passes into the glomerular filtrate and is reabsorbed in the proximal tubules, only a small amount of B2M can be detected in the urine under normal conditions. Therefore, increased urine B2M levels strongly suggest proximal tubule injury [2]. Cystatin C is an early biomarker of CKD and its levels are less affected by clinical characteristics, such as gender, age, body size and composition. It is superior to GFR calculated from creatinine [16]. LCN-2 has been established as an early biomarker of AKI. Prior to an increase in serum creatinine levels, an increase in urine LCN-2 levels could be detected in response to AKI. LCN-2 is not only an important predictor of AKI, disease progression, and mortality but also helps to differentiate between AKI aetiologies [17].

Our findings support the concept that B2M is related to stroke risk. A study revealed that high levels of B2M were associated with an increased risk of ischemic stroke among women [18], which is consistent with our findings. Previous studies have found a significant association of B2M with CVD. The Atherosclerosis Risk in Communities (ARIC) Study showed that a more than 30% decline in 1/B2M was related to increased risk of incident CVD (HR: 1.79; 95% CI: 1.36, 2.34) in US adults after a 14-year median follow-up period [19]. In the Chronic Renal Insufficiency Cohort (CRIC) Study, B2M was independently associated with CVD in US adults with mild-to-moderate CKD [6]. Of 2405 participants without CVD at baseline, 292 developed CVD over a six-year median follow-up. It was found that 1/B2M had a significant association with CVD, and it remained significant after adjusting for age, sex, race and other confounders (HR: 1.45; 95% CI: 1.22, 1.72) [6]. Recently another meta-analysis involving 30,988 participants and 5391 CVD events revealed that compared to the lowest tertile of B2M levels, the highest tertile was associated with higher risk of CVD (RR:1.71; 95% CI: 1.37, 2.13) and stroke (RR:1.51; 95% CI: 1.28, 1.78) [20]. The majority of previous studies focused on the relationship between B2M and CVD. The current literature on the relationship between B2M and stroke is still limited. Our study highlights the significant association between B2M and stroke risk in the general population.

Cystatin C was significantly associated with stroke risk in our study. The role of cystatin C in stroke has been controversial. Our results are consistent with those in the China Health and Retirement Longitudinal Study [21] and Cardiovascular Health Study [22]. In the China Health and Retirement Longitudinal Study reported recently, 410 (5.8%) out of 7064 participants developed new-onset stroke after 7 years of follow-up. Cystatin C was associated with new-onset stroke (HR: 1.19; 95% CI: 1.14, 1.25) [21], thereby confirming our findings. In the Cardiovascular Health Study, cystatin C was significantly associated with incident ischemic stroke and was a marker of poor outcome (shorter survival, cognitive decline and activities of daily living decline) after ischemic stroke in old adults [22]. However, conflicting results have been reported in other studies. A Mendelian randomization study using participant data from 16 prospective cohorts showed that cystatin C was not causally associated with CVD (RR: 1.00; 95% CI: 0.82, 1.22) and stroke (RR: 0.82; 95% CI: 0.57, 1.18) [9]. Thus, it seems that cystatin C is more a marker than a cause of vascular disease, although in animal models, cystatin C plays a role in endogenous neuroprotection and helps to maintain lysosomal membrane integrity [10]. Our study confirms the significant association between cystatin C and stroke, but further studies are needed to elucidate the mechanisms involved.

With regard to LCN-2, our data suggest a significant association with stroke risk. The role of LCN-2 in the risk of stroke has been investigated in patients with AKI and CKD. A previous Chinese study recruited 205 patients (40 stroke patients with AKI and 165 stroke patients without AKI) and 80 healthy controls. It revealed that the concentration of serum LCN-2 increased with the severity of stroke in patients with AKI [12]. A Japanese study reported that among 252 CKD patients at baseline, 36 CVD events (5 stroke events) occurred after a median 63-month follow-up period. Plasma LCN-2 was found to be associated with CVD (HR:1.005; 95% CI: 1.003, 1.007) [13]. Our study has a larger sample size and can therefore reveal the significant association between LCN-2 and stroke risk in the general population. In the Prevention of Renal and Vascular End-stage Disease study, a non-linear relationship was observed between LCN-2 and the risk of CVD, but LCN-2 was not associated with stroke in this study in Caucasians [23]. The apparent disagreement with our study may be related to population characteristics. Strokes are more common in Chinese. Our study of Chinese adults, therefore, had more power to reveal a significant association between LCN-2 and stroke risk.

Stroke and coronary heart disease (CHD) are both vascular diseases and so they share similar pathophysiological mechanisms. B2M, lipocalin-2 and cystatin C have been shown to be associated with CHD [20,24,25]. CHD is also known to be associated with cognitive deficits [26]; in a large British cohort, episodic memory, semantic verbal fluency, fluid reasoning and numerical ability were inversely associated with CHD. Previous studies of polymorphisms did not provide evidence that cystatin C plays a role in the aetiology of CHD [9,27,28]. A single-nucleotide polymorphism (SNP), rs13297295, has been found to be associated with elevated levels of LCN-2 and cardiac hypertrophy [29]. Our research team previously found an association of haplotypes in LCN2 with elevated blood pressure [30].

The possible pathophysiological mechanisms between these three renal biomarkers and stroke have been reported previously. B2M stabilized the surface expression of MHC-I and other members of the MHC-I family, which play an important role in both the innate and adaptive immune systems [31]. As a trigger for the inflammatory process, B2M is related to atherosclerosis, which underlies the development of stroke [32,33]. Cystatin C has a direct effect on the balance of extracellular matrix proteins interacting with vessel wall remodelling [34]. High cystatin C concentration was found to be involved in inflammation, which promotes atherosclerosis [35]. LCN-2 may contribute to the development of atherosclerotic plaques by interacting with matrix metalloproteinase-9 [36]. Our previous study found that the accumulation of deamidated lipocalin-2 in arteries caused vascular inflammation and endothelial dysfunction, which are key processes in the development of stroke [37]. LCN-2 and B2M correlate with oxidative stress [38,39], which plays an important role in the pathogenesis of ischaemic stroke. The cellular effects of oxidative stress in ischaemic stroke include lipid peroxidation, protein denaturation, inactivation of enzymes, nucleic acid and DNA damage, which lead to neuronal damage and neuronal death [40]. Glial scar formation could be detected after stroke [41]. These findings provide a pathophysiological basis for the association of these three biomarkers with stroke.

This is a cross-sectional study, so a causal relationship between the renal biomarkers and stroke could not be inferred. Moreover, a larger sample size is needed for reliable subgroup analysis. Despite these limitations, we found evidence of association between renal biomarkers and stroke risk in the Chinese population.

## Conclusion

Serum B2M, cystatin C and LCN-2 are significantly associated with stroke risk and are potential novel clinical biomarkers in the assessment of stroke risk.

## Data Availability

The data that support the findings of this study are available from the corresponding author upon reasonable request.
